# Quality evaluation of commercial ‘Boju’ (*Chrysanthemum morifolium* Ramat.) using integrated electronic nose, electronic tongue, and GC-IMS analysis

**DOI:** 10.3389/fnut.2026.1945038

**Published:** 2026-10-23

**Authors:** Xiao-su Xiang, Yun-yun Sun, Jun-shan Gao, Da-hui Li, Qing-shan Yang

**Affiliations:** 1 College of Pharmacy, Anhui University of Chinese Medicine, Hefei, China; 2 School of Life Sciences, Anhui Agricultural University, Hefei, China; 3 Anhui Province Key Laboratory of Research & Development of Chinese Medicine, Hefei, China; 4 Institute of Conservation and Development of Traditional Chinese Medicine Resources, Anhui Academy of Chinese Medicine, Hefei, China; 5 Synergetic Innovation Center of Anhui Authentic Chinese Medicine Quality Improvement, Hefei, China

**Keywords:** ‘Boju’, chemometrics, electronic nose, electronic tongue, gas chromatography—ion mobility spectrometry

## Abstract

Commercial ‘Boju’ (*Chrysanthemum morifolium* Ramat.), derived from the dried capitulum of Asteraceae, is a medicinal herb of significant value. Currently, two primary commercial specifications of ‘Boju’ are circulated in the market: *Chrysanthemum morifolium* Hemsl cv. ‘Da Boju’ (DBJ) and *Chrysanthemum morifolium* Hemsl cv. ‘Xiao Boju’ (XBJ). Their original plant sources are *Chrysanthemum × morifolium* Hemsl cv. *‘Damaya’*. and *Chrysanthemum × morifolium* Hemsl cv. *‘Boju’*, respectively. ‘Boju’, is a globally consumed functional herb valued for its distinctive organoleptic properties. To address the challenge of cultivar-specific authentication, an integrated multi-sensor platform synergizing electronic nose (E-nose), electronic tongue (E-tongue), and gas chromatography-ion mobility spectrometry (GC-IMS) was deployed. While E-nose profiles effectively segregated DBJ from XBJ, e-tongue analysis revealed largely overlapping sensor responses across both groups, with saltiness-like and umami-like channels predominating; DBJ was primarily distinguished by significantly stronger bitter-aftertaste responses. GC-IMS metabolomics resolved 125 volatile signals, enabling the annotation of 66 compounds; subsequent multivariate modeling isolated 28 terpenoid—enriched biomarkers that underpinned the observed aromatic divergence. This non-destructive data fusion framework offers a robust paradigm for the quality grading and standardization of chrysanthemum-based functional foods.

## 1 Introduction

The conventional paradigm for assessing food quality has long involved a dual approach of sensory panels and physicochemical assays. However, the advent of bionic sensing technologies—specifically the E-nose and E-tongue—has introduced a shift toward simulating human perception for the rapid, objective characterization of flavor profiles (1, 2). By mitigating the inherent subjectivity and variability of manual evaluations, these intelligent systems excel at discerning subtle distinctions across diverse food matrices (3, 4). Concurrently, gas chromatography-ion mobility spectrometry (GC-IMS) has emerged as a formidable technique for volatile analysis. Its capacity to detect trace compounds at the ppb level, coupled with a rapid detection cycle (10–15 min per sample), offers distinct advantages over conventional GC-MS in constructing visual fingerprints of volatile components (5–7).

The strategic amalgamation of these intelligent sensors with GC-IMS, particularly when leveraged through multivariate chemometrics (e.g., PCA, OPLS-DA), enables a holistic deconstruction of food attributes spanning both sensorial perception and molecular composition (8). This multi-modal data fusion architecture has substantiated its discriminatory robustness in authenticating high-value medicinal plants, such as *Gastrodiae* Rhizoma (9), and *Atractylodis* Rhizoma (10), highlighting its potential for standardized quality control.

Within the domain of functional florals, ‘Boju’ represents a preeminent cultivar among China’s five major medicinal-edible homologous resources, endemic to Bozhou, Anhui Province. Revered as a staple herbal infusion, it is canonically acknowledged for its therapeutic potentials, including dispelling wind-heat and ameliorating ocular health (11). Revious studies have demonstrated that DBJ and XBJ differ in both the types and contents of their chemical constituents. Although the phytochemistry of its bioactive constituents (e.g., flavonoids and phenolic acids) is well-documented, a comprehensive elucidation of its flavorome—particularly from a food consumption perspective—remains nascent. The current marketplace is bifurcated into two predominant commercial specifications: DBJ and XBJ. These cultivars manifest significant divergence in morpho-sensory phenotypes, critically dictating consumer palatability and economic valuation. Nevertheless, systematic investigations into their flavoromic divergence are conspicuously absent. Consequently, this study employed an integrated multi-sensor analytical platform—combining E-nose, E-tongue, and GC-IMS—to compare the flavor profiles of commercially available DBJ and XBJ. Rather than isolating cultivar-intrinsic differences under controlled conditions, this work evaluates sensory variations among commercial batches as encountered by consumers, providing practical insight into the flavor distinctions between these two commercially important chrysanthemum products.

## 2 Materials and methods

### 2.1 Samples and reagents

Three independent commercial batches of DBJ (*Chrysanthemum × morifolium* (Hemsl) Hemsl. *‘Damaya’* cv. nov.) and XBJ (*Chrysanthemum × morifolium* (Hemsl) Hemsl. *‘Boju’* cv. nov.) were purchased from different vendors at the Bozhou Medicinal Materials Market, Anhui Province, China, in December 2025. Each batch was assigned a unique code (DBJ-1 to DBJ-3; XBJ-1 to XBJ-3). All samples were sold as dried capitula; however, vendors did not provide complete information regarding primary processing methods (e.g., shade drying, hot-air drying, or possible sulfur fumigation), and therefore the exact drying and post-harvest treatment history of each batch could not be verified. Samples were authenticated by Associate Professor Yang Qingshan (Anhui University of Chinese Medicine) as the respective cultivars based on morphological examination. It should be noted that, because samples were commercially sourced and processing histories were not fully documented, observed differences between DBJ and XBJ reflect variations among commercial batches and may be influenced by factors including cultivar., vendor-specific processing, storage conditions, or post-harvest treatments, rather than cultivar alone. Dried DBJ and XBJ samples (medicinal material) were collected from markets, immediately sealed in food-grade polyethylene bags, and stored at −20 °C in the dark (wrapped with aluminum foil) until analysis. Prior to each experiment, samples were pulverized using a traditional Chinese medicine grinder (Beijing Zhaoshen Technology Co., Ltd., China), passed through a No. 4 pharmacopoeial sieve (nominal aperture: 250 μm), and the resulting powder was resealed in polyethylene bags and stored at 4 °C in the dark for 24 h before electronic nose, electronic tongue, and GC-IMS analyses.

Tartaric acid, potassium chloride, concentrated hydrochloric acid, potassium hydroxide, silver chloride, and n-ketones (2-butanone, 2-pentanone, 2-hexanone, 2-heptanone, 2-octanone, and 2-nonanone; all of analytical grade) were purchased from Shanghai Aladdin Biochemical Technology Co., Ltd. Ultrapure water was used throughout the experiments.

### 2.2 Electronic nose analysis methods

Pen3 Plus electronic nose (Airsense Analytics GmbH, Schwerin, Ger many) contains ten oxide sensors (receptors) is also known as receptors, each of which exhibits sensitivity to a distinct group of compounds. The following sensors are sensitive to various chemical compounds: sensor W1C is selective to benzene-like and aromatic compounds; sensor W5S is sensitive to nitrogen oxides; sensor W3C is selective to ammonia and aromatic compounds; sensor W6S is selective to hydrides; sensor W5C is sensitive to short-chain alkanes and compounds that contain an aromatic ring; sensor W1S is reactive to short-chain alkanes, such as methane; sensor W1W is responsive to inorganic sulfur compounds; sensor W2S is sensitive to alcohols, ethers, aldehydes, and ketones; sensor W2W is selective to aromatic compounds and other sulfur chemicals; and finally, sensor W3S is able to detect long-chain alkanes. An aliquot of 3 g of sample powder was accurately weighed, placed into a dedicated measuring cup, and sealed with Parafilm. The sealed cup was then left to stand overnight under constant temperature conditions to allow the generation of stable volatile compounds, after which the measurement was performed. Each sample was measured in triplicate (12).

### 2.3 Electronic tongue analysis methods

The taste characteristics of DBJ and XBJ were evaluated using an electronic tongue instrument (SA402BPlus - EX, Insent, Japan). The evaluated taste attributes included sourness, sweetness, bitterness, umami, saltiness, astringency, bitter aftertaste, astringent aftertaste, and richness (umami aftertaste). A 1.5 g aliquot of sample powder (passed through a No. 4 pharmacopoeial sieve, nominal aperture 250 μm) was accurately weighed and transferred into a 250 mL stoppered conical flask. Distilled water (100 mL) was added, and the mixture was thoroughly agitated to ensure complete wetting of the powder. Ultrasonic extraction was performed using a KQ-400VSM ultrasonic cleaner (Kunshan Ultrasonic Instrument Co., Ltd., China) at 100 W and 40 kHz for 30 min at room temperature (≈ 25 °C). The flask was sealed with a stopper throughout the extraction process. After cooling to room temperature, the extract was transferred into 10 mL centrifuge tubes and centrifuged at 2800 rpm (approximately 655 xg, rotor radius ~7.5 cm) for 5 min at room temperature using an H4-20K high-speed centrifuge (Hunan Kecheng Instrument Equipment Co., Ltd., China) equipped with a 12 × 10 mL angle rotor (No. 4). The supernatant was then filtered through a 0.45 μm nylon 66 syringe filter (13 mm diameter, SCIDISC, Cat. No. S13NY-045) using a disposable syringe. The filtrate was subsequently transferred into a dedicated sample vessel for electronic tongue analysis. The filtrate was subsequently transferred into a dedicated sample vessel for electronic tongue analysis. Each sample was prepared in duplicate, and the measurement was cycled four times. Data from the last three cycles were used for subsequent analysis (13).

### 2.4 GC-IMS analysis methods

The GC-IMS analysis was executed through the utilization of an MXT-5 column (15 m × 0.53 mm, 1.0 μm), an autosampler unit (CTC Analytics AG, Zwingen, Switzerland), and an IMS instrument (FlavourSpec®, G. A. S., Dortmund, Germany). The precise weight of 2.0 g of the specimen was meticulously calculated and transferred into a 20 mL sterile headspace vial, with three replicates being prepared for each specimen. An internal standard consisting of a 10 μL aliquot of 10 ppm 4-methyl-2-pentanol solution was incorporated into each vial. The incubation conditions employed for the headspace experiment were as follows: the temperature was set to 80 °C, the duration of theubation was 20 min, the amount of liquid injected was 200 μL, the injection was done without a split, the agitation speed was set to 500 r/min, and the temperature of the injection needle was set to 85 °CThe GC conditions are outlined as such: initial column temperature of 60 °C; carrier gas of high-purity nitrogen with a minimum purity of 99.999%; a programmed pressure rampe, with the following initial flow rate of 2.0 mL/min for a period of 2 min, followed by an eight-minute period during which the flow rate increased linearly to 10.0 mL/min, then 10 min of linear increment to 100.0 mL/min. This was followed by10 min of constant 150.0 mL/min, concluding with a final ten-minute holding period. The GC runtime was recorded at 40 min, and the injector temperature was maintained at 80 °C. The IMS conditions were as follows: ionization source tritium (^3^H); drift tube length 53 mm; electric field strength 500 V/cm; drift tube temperature 45 °C; drift gas high-purity nitrogen (purity ≥ 99.999%) at a flow rate of 75 mL/min; positive ion mode (14).

A qualitative evaluation of the target molecules was conducted through retrieval and comparison with the GC retention index (NIST 2020) database and the IMS drift time database, which were integrated within the VOCal software. In addition,a comparative assessment of the volatile organic compounds present in the diverse samples was executed by leveraging specialized plugins, namely Reporter and Gallery Plot, within the VOCal data processing platform.

### 2.5 Statistical analysis

To this end, professional statistical analysis tools were leveraged to conduct a thorough and methodical examination of the volatile compounds in question. Radar plot visualization was performed using Origin 2023 software (OriginLab Corporation, Northampton, MA, USA), while principal component analysis (PCA) and orthogonal partial least squares discriminant analysis (OPLS-DA) were conducted using SIMCA 14.1 software (Umetrics, Umea, Sweden). Data processing for multivariate analysis: for PCA and OPLS-DA, data from three parallel samples within each batch were averaged to obtain one representative value per independent batch (*n* = 3 batches per variety, total *n* = 6). This approach avoids pseudoreplication by ensuring each point in multivariate space represents an independent collection batch. For univariate analysis (bar charts, *t*-tests), all parallel measurements were used (*n* = 9 per variety) to increase statistical power, with data reported as mean ± SD. OPLS-DA models were validated by 7-fold cross-validation and 200 permutation tests to assess the risk of overfitting. Independent samples *t*-tests were performed using SPSS 26.0 (IBM Corp., Armonk, NY, USA), with *p* < 0.05 considered statistically significant (15).

## 3 Results

### 3.1 E-nose analysis

The electronic nose responses of commercially available DBJ and XBJ were evaluated using both radar and bar charts (Figures 1, 2). The radar plot revealed distinct overall response patterns, with sensors W1W and W2W showing high values, particularly in DBJ, while W6S exhibited a markedly higher response in XBJ.

**Figure 1:**
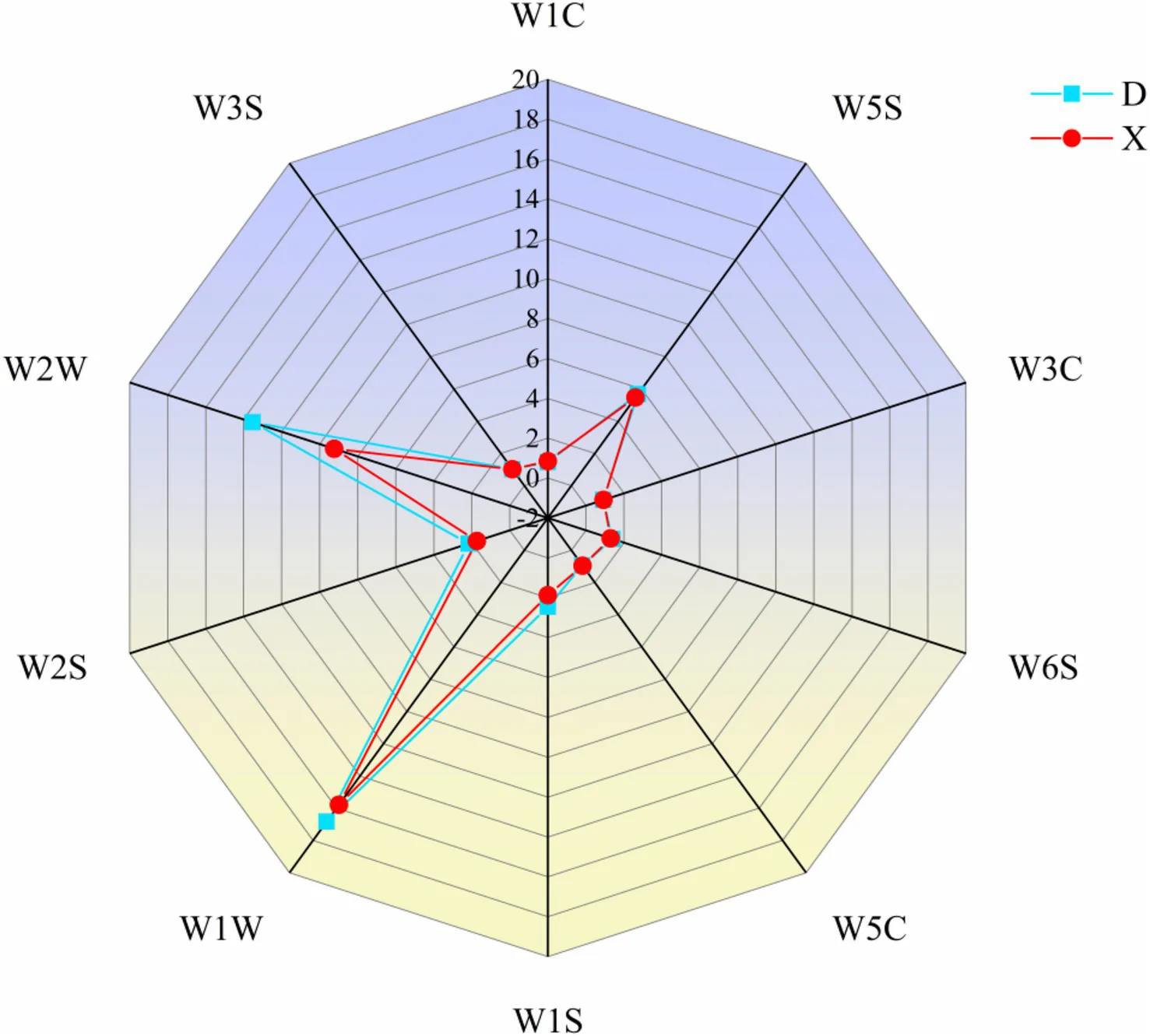
Radar plot of E-nose responses.

**Figure 2:**
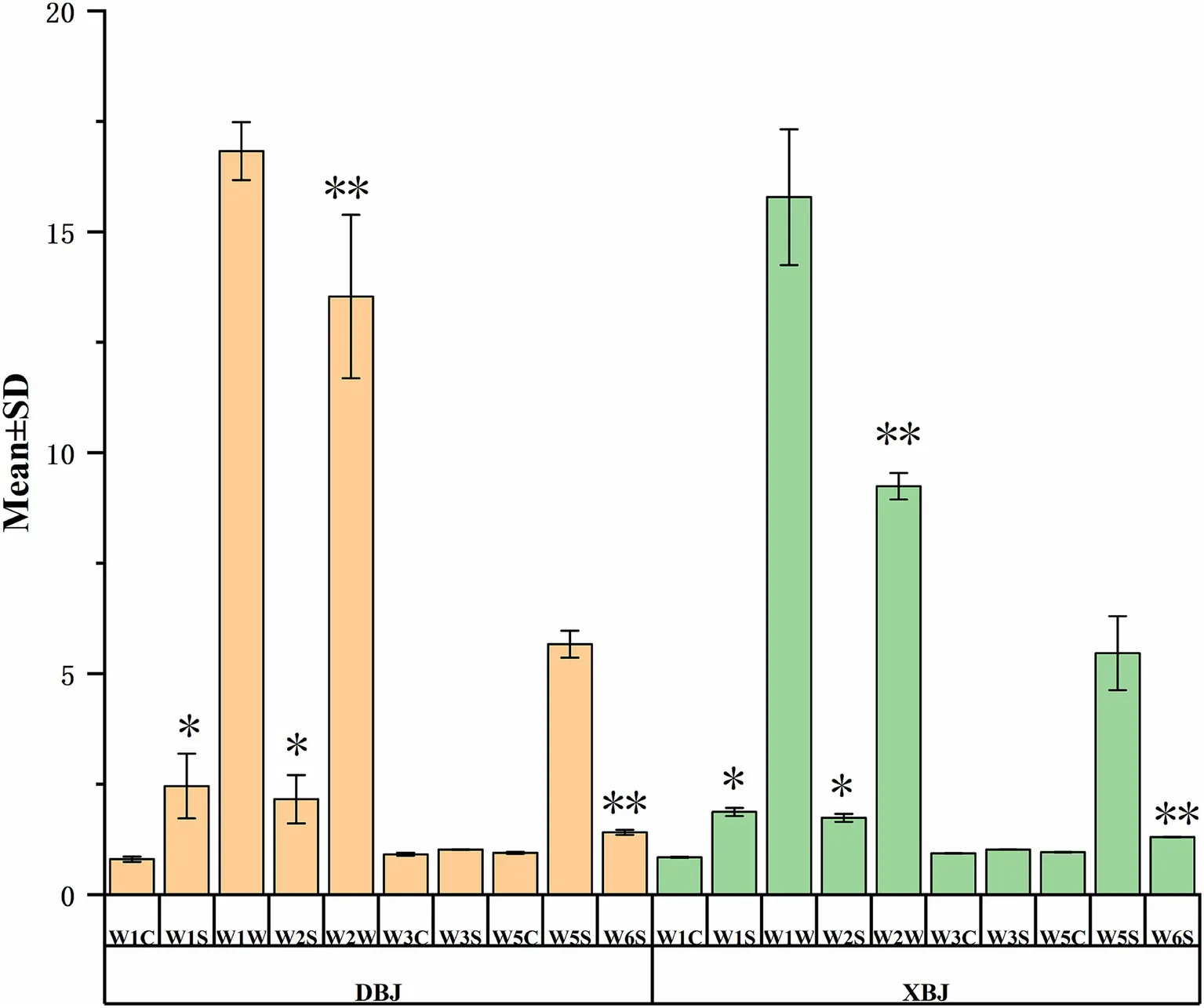
E-nose responses of DBJ and XBJ (mean ± SD, *n* = 9). *p < 0.05, **p < 0.01 (independent samples *t*-test; horizontal lines denote intergroup significance).

Quantitative statistical analysis (independent samples *t*-test, *n* = 9) with mean ± SD error bars demonstrated significant intergroup differences. Specifically, DBJ showed significantly higher responses in W2W (*p* < 0.01), W1S (*p* < 0.05), and W2S (*p* < 0.05), whereas XBJ exhibited a significantly higher response in W6S (**p < 0.01). No statistically significant difference was observed for W1W (*p* > 0.05). These results indicate that DBJ and XBJ differ mainly in sulfur-containing compounds, aromatic compounds, and nitrogen oxides, representing key odor-active volatile components of ‘Boju’, supported by appropriate variability and statistical testing (16).

In the following section, a detailed procedure is outlined to facilitate a more comprehensive analysis of the olfactory distinctions between DBJ and XBJ. The set of response measurements from the ten electronic nose sensors was subsequently entered into SIMCA 14.1 for PCA analysis. As demonstrated in the PCA score plot (Figure 3A), the first principal component contributed 56.1% to the total variance, the second principal component contributed 25.6%, and the cumulative percentage of variance explained was 81.7%. This finding suggests that the utilization of principal components effectively captures the inherent structure of the original data. Consequently, a clear distinction in odor could be discerned among commercially available DBJ and XBJ samples through the analysis of signal values derived from electronic nose sensors.

**Figure 3:**
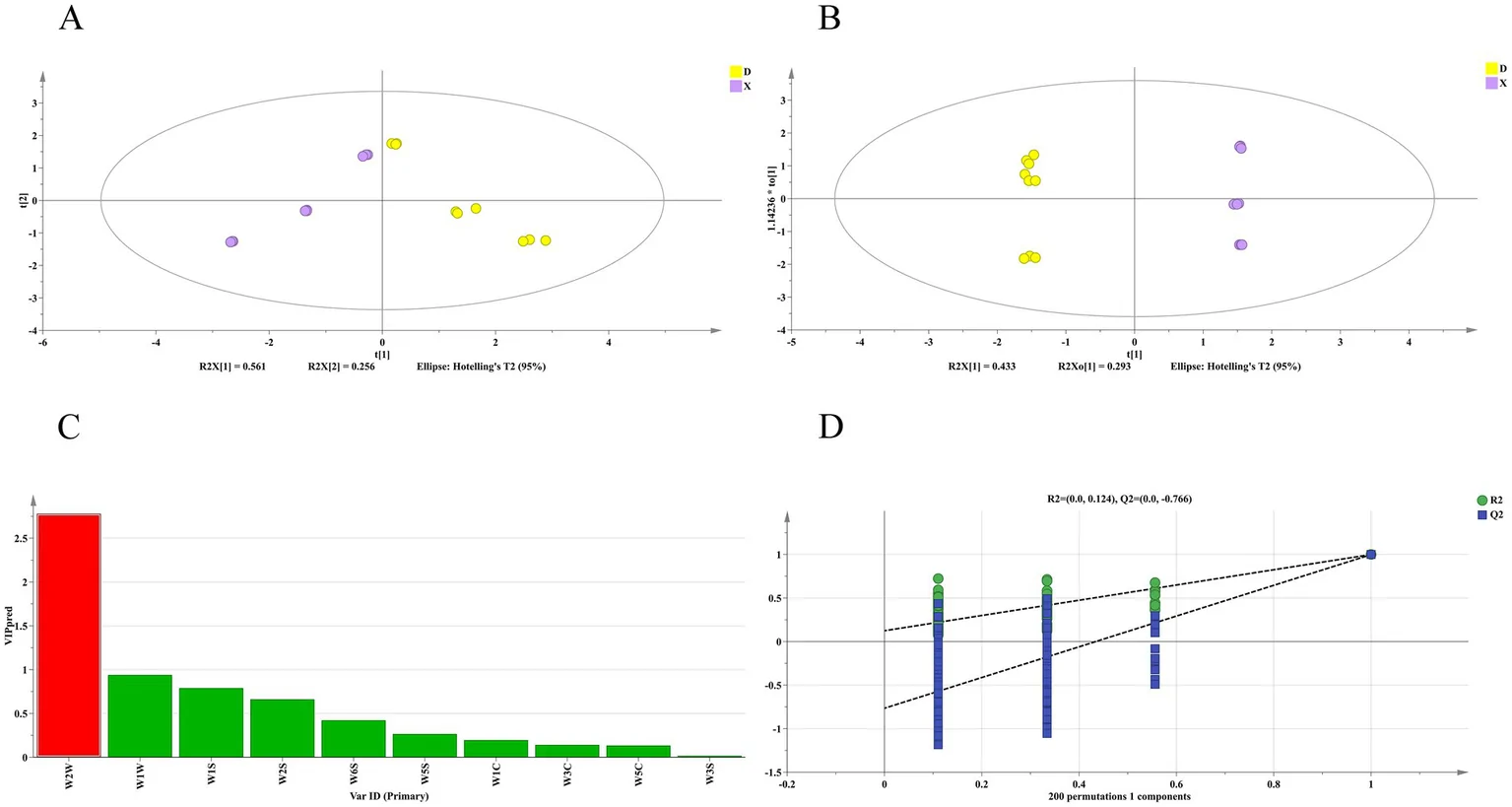
Chemometric analysis of E-nose sensor data: **(A)** PCA score plot. **(B)** OPLS-DA score plot. **(C)** VIP plot of 24 common peaks. **(D)** Permutation test plot with 200 permutations.

In order to ascertain which compounds are responsible for the olfactory discrepancies between DBJ and XBJ, OPLS-DA was conducted on the E-nose sensor signal values. The corresponding score plot is presented in Figure 3B. DBJ and XBJ were well separated into two distinct groups, which is in alignment with the results obtained through PCA. The variable importance in projection (VIP) value is a critical metric for assessing the significance of each OPLS-DA model variable. According to the screening criterion of VIP value > 1, the W2W sensor was identified as having a relatively large contribution to the discrimination between DBJ and XBJ, indicating that aromatic compounds and organic sulfur compounds may significantly influence sample classification and are the main potential marker components driving the odor discrepancies between the two varieties. The results of this study are presented in Figure 3C. A permutation test with 200 iterations was conducted on the OPLS-DA model. As demonstrated in Figure 3D, the intercept of the Q^2^ regression curve with the ordinate axis was negative, suggesting that the model was not overfitted and validating its reliability. Consequently, this model can be utilized to differentiate between the odor profiles of DBJ and XBJ.

### 3.2 E-tongue analysis

The radar plot (Figure 4) and bar chart (Figure 5) illustrate the E-tongue taste responses of commercial DBJ and XBJ. As shown in Figure 4, both samples exhibit relatively high sensor response values for sweetness-like, umami-like, bitterness-like, and richness-like channels, indicating these traits predominantly characterize their taste profiles.

**Figure 4:**
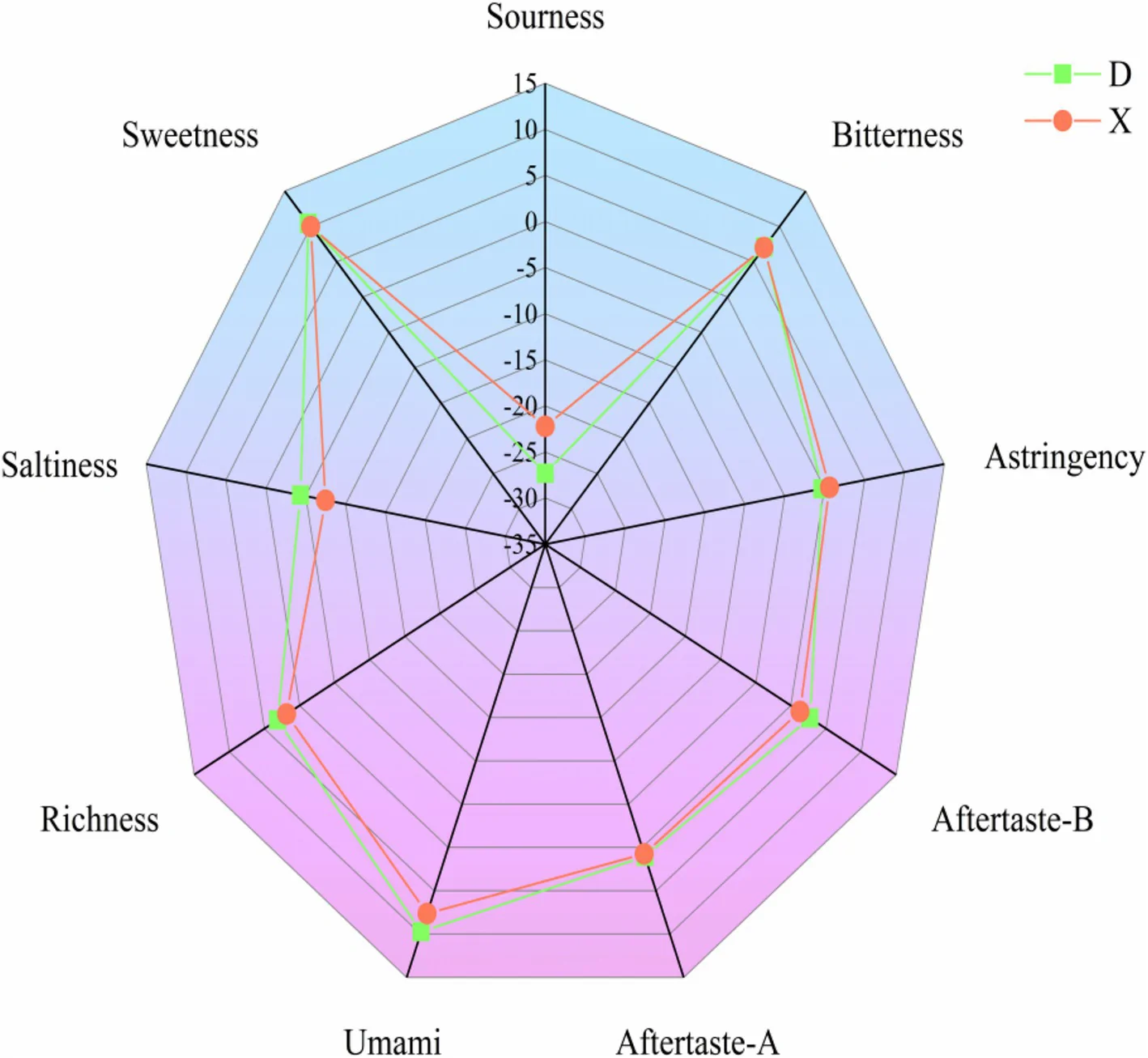
Radar plot of taste responses obtained by E-tongue.

**Figure 5:**
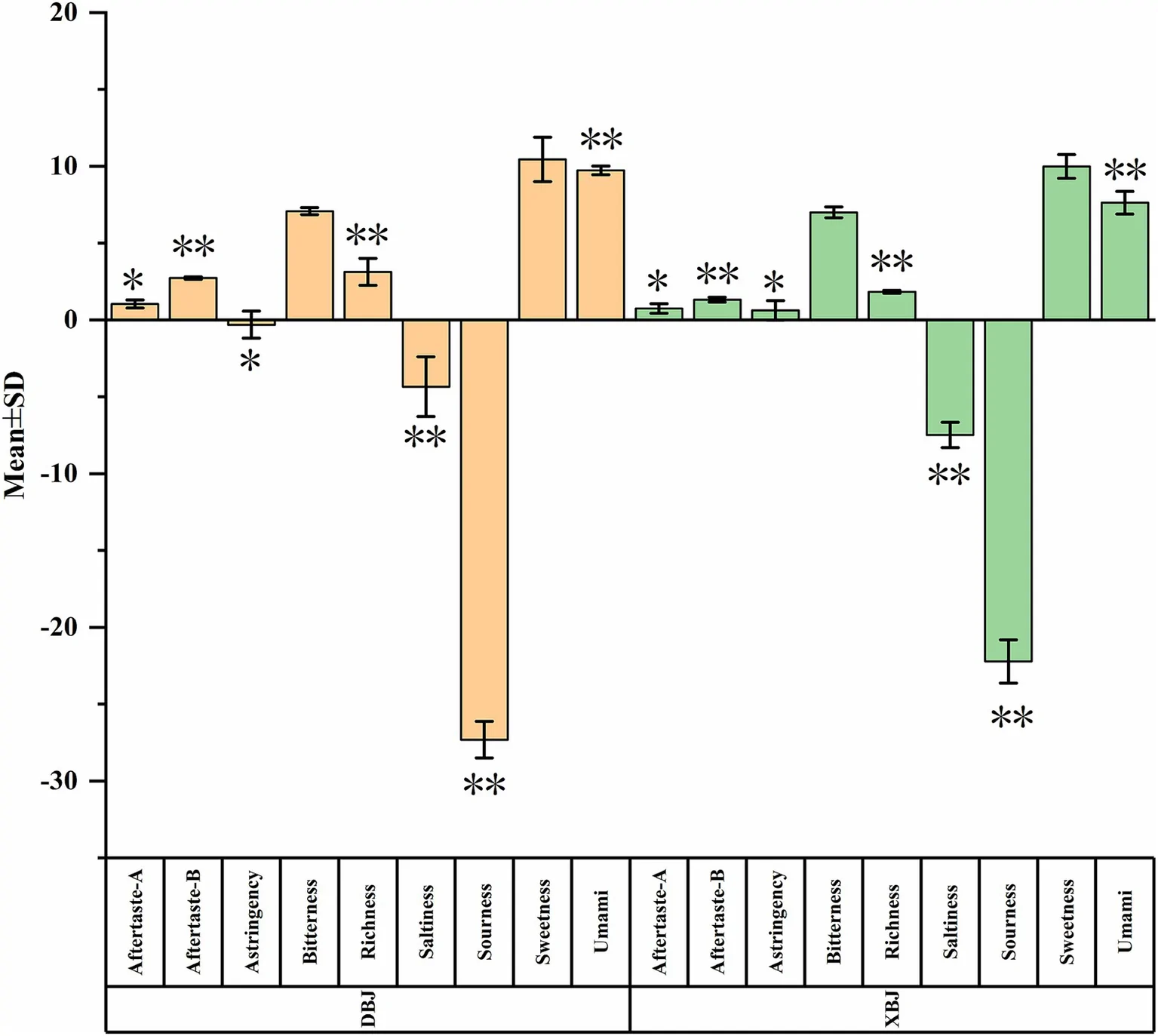
E-tongue responses of DBJ and XBJ (mean ± SD, *n* = 9). * *p* < 0.05, ** *p* < 0.01 (independent samples t-test; horizontal lines denote intergroup significance).

Contrary to the original assumption, the sourness sensor response was not minimal but showed extreme negative values (DBJ: -27.31, XBJ: −22.22), with DBJ exhibiting a significantly stronger sour response (*p* < 0.01, Figure 5). For the saltiness channel, XBJ (−7.48) was significantly lower than DBJ (−4.34) (*p* < 0.01), suggesting a weaker saltiness-like sensor response in XBJ, while DBJ showed a relatively stronger response in this channel.

Significantly higher responses in DBJ compared to XBJ were also observed for umami (*p* < 0.01), richness (*p* < 0.01), bitter aftertaste-A (*p* < 0.05), and bitter aftertaste-B (*p* < 0.01). Conversely, astringency was significantly higher in DBJ (*p* < 0.05), while sweetness-like and bitterness-like channels showed no significant differences (*p* > 0.05) between the two groups, consistent with the overlapping areas in the radar plot. Overall, DBJ demonstrated more intense and differentiated sensor responses across sourness, saltiness-like, umami, richness, and aftertaste channels, whereas XBJ was primarily distinguished by its extreme negative sourness response and relatively attenuated secondary sensor intensities (17).

In order to undertake a more comprehensive analysis of the gustatory distinction between DBJ and XBJ, the response values acquired from the electronic tongue were integrated into SIMCA 14.1 for PCA. As can be seen from the PCA score plot (Figure 6A), the initial principal component demonstrated a contribution rate of 63.4%, that of the second principal component was 19.1%, and the cumulative contribution rate was 82.5%, indicating that the principal components could adequately reflect the characteristics of the original data. Therefore, the taste of commercially available DBJ and XBJ could be clearly distinguished using the electronic tongue response values.

**Figure 6:**
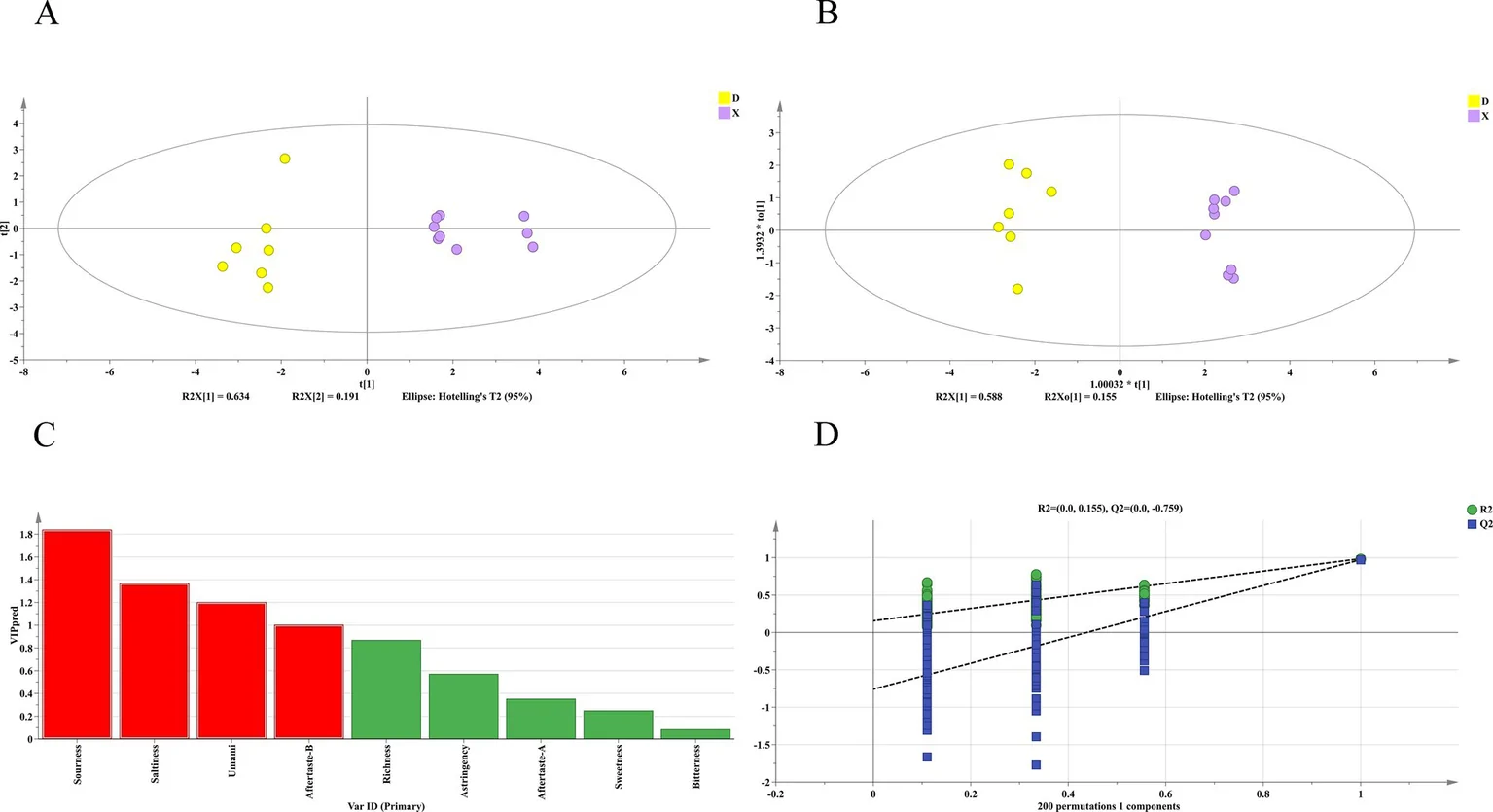
Chemometric analysis of E-tongue measurements: **(A)** PCA score plot. **(B)** OPLS-DA score plot. **(C)** VIP plot of 24 common peaks. **(D)** Permutation test plot with 200 permutations.

In order to ascertain which taste attributes are responsible for the taste disparities between DBJ and XBJ, OPLS-DA was performed on the response values measured by the electronic tongue. The corresponding score plot is presented in Figure 6B. DBJ and XBJ were well separated into two distinct groups, which is consistent with the PCA results. The VIP value is an important indicator for evaluating the contribution of each variable in the OPLS-DA model; the detailed VIP values are presented in Table 1. According to the screening criterion of VIP value > 1, sourness, saltiness, umami, and bitter aftertaste were identified as having relatively large contributions to the discrimination between DBJ and XBJ. However, the response value for sourness was below the taste zero point (− 13) and thus was considered of no reference value. The saltiness response value of XBJ (− 7.48) was below its taste zero point (− 6), indicating no or extremely weak saltiness, whereas that of DBJ (− 4.34) was above the taste zero point, indicating a slight saltiness. The findings suggest that saltiness, a distinctive attribute of DBJ, along with umami and bitter aftertaste, exert a substantial influence on the classification of the samples. These taste attributes appear to be the primary markers responsible for the observed differences in taste between the two varieties, as illustrated in Figure 6C. A permutation test comprising 200 iterations was performed on the OPLS-DA model. As demonstrated in Figure 6D, the intercept of the Q^2^ line of best fit with the vertical axis was found to be negative, a finding that indicates the absence ofover fitting and thereby substantiating the validity of the model. Subsequently, this model can be used to discriminate the taste differences between DBJ and XBJ. In consequence, the E-tongue can distinguish between commercially available DBJ and XBJ.

**Table 1:** E-tongue sensor VIP scores.

Var ID (Primary)	VIP pred
Sourness	1.84036
Saltiness	1.36991
Umami	1.20294
Aftertaste - B	1.00416
Richness	0.870301
Astringency	0.572552
Aftertaste - A	0.354277
Sweetness	0.250796
Bitterness	0.0858066

### 3.3 GC-IMS analysis

A recently developed GC-IMS technique was utilized to further investigate the distinctions in volatile compounds between commercially available DBJ and XBJ. This technique achieves efficient separation based on the differential drift velocities of ionized volatile organic compounds in a weak electric field, i.e., differences in their arrival times at the detector after colliding with a counterflow drift gas. Using this approach, qualitative and semi-quantitative analyses of volatile organic compounds in the two varieties of ‘Boju’ were performed, and their aroma characteristics were systematically evaluated.

#### 3.3.1 Comparative analysis of GC-IMS spectra

In order to provide a thorough characterization of the volatility of the organic compounds contained within DBJ and XBJ, three-dimensional (3D) spectra, two-dimensional (2D) fingerprint maps, and respective difference plots were generated using GC-IMS (Figures 7A–C). As shown in Figure 7A, the X-axis is indicative of drift time, the Y-axis corresponds to retention time, and the Z-axis represents signal peak amplitude. Spectral analysis reveals discernible variations in volatile organic compounds between DBJ and XBJ. For further visualization, Figure 7B presents the top view of the 3D spectra. The underlying context of the entire narrative is set against a blue background, and the red line that emerges along the x-axis at 1.0 a.u. corresponds to the normalized reagent ion peak (RIP), as determined by the analytical process. The presence of a volatile organic compound can be indicated by the observation of a specific punto on opposing sides of the RIP peak. The color of the indicator is indicative of the peak intensity of the compound in question, where darker colors correspond to greater peak intensities, ranging from blue to red. As illustrated in Figure 7B, following a relative drift time of approximately 1.5 a.u., the peak intensities of volatile compounds in XBJ exceed those in DBJ. In order to make a more direct and apples-to-apples comparison of the discrepancies in volatile components between DBJ and XBJ, the spectrum of the DBJ sample was utilized as a benchmark, by subtracting the XBJ spectrum from the reference to generate a difference comparison plo, as demonstrated in Figure 7C. In the event that the content of a volatile organic compound is equivalent in the sample under consideration and the designated reference sample, the resultant difference will appear white. Red indicates elevated concentrations of the compound in the target sample relative to the reference sample. Conversely, blue signifies reduced concentrations of the compound in the target sample compared to the reference sample. As indicated in Figures 7C, a discernible divergence in the composition of volatile compounds is apparent between the DBJ and XBJ samples (18).

**Figure 7:**
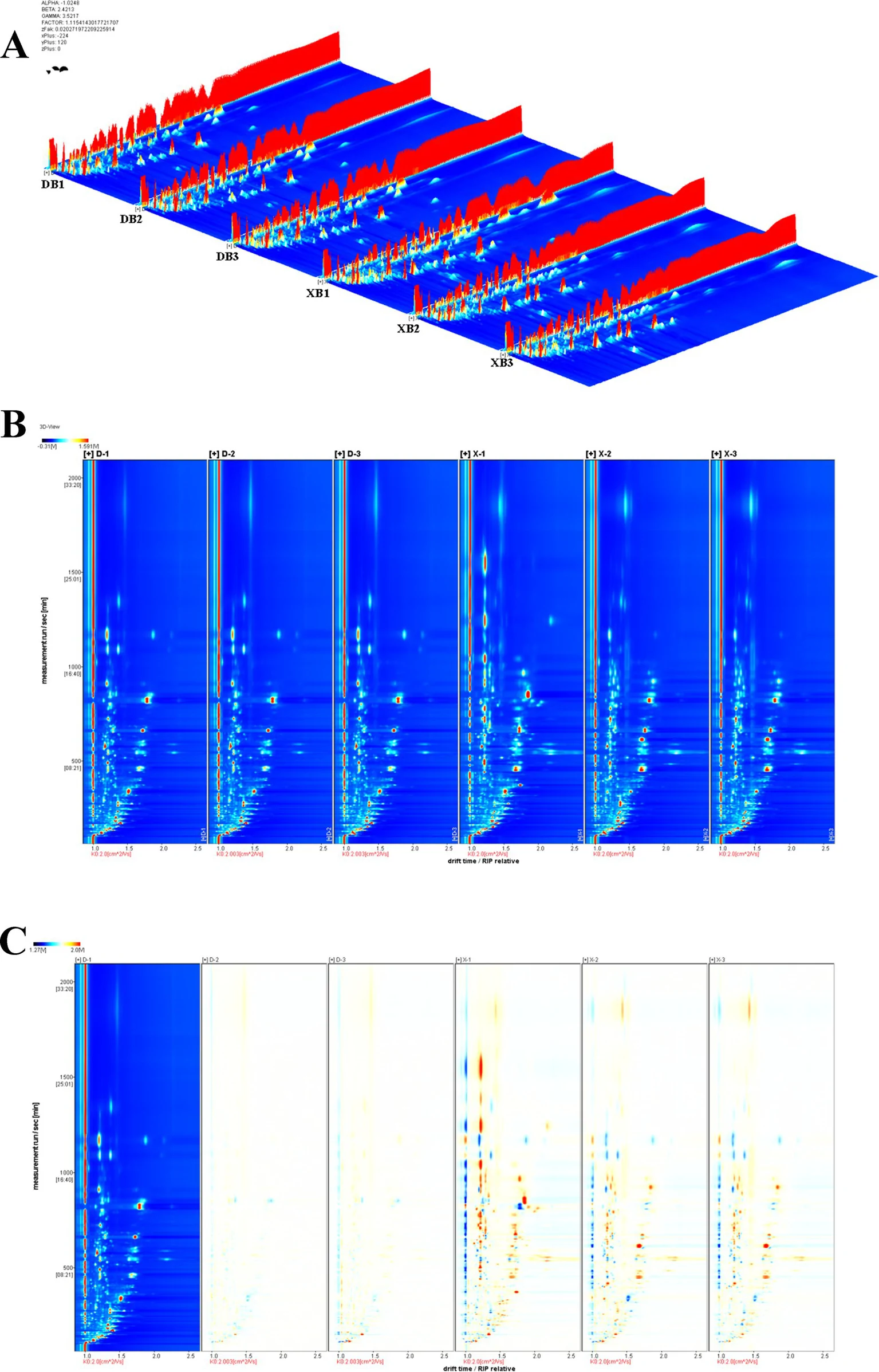
GC-IMS spectra of volatile components in samples: **(A)** Three-dimensional GC-IMS spectrum of volatile components in samples; **(B)** Two-dimensional GC-IMS spectrum of volatile components in samples; **(C)** Differential GC-IMS spectrum of volatile components in samples.

#### 3.3.2 Qualitative analysis and characteristic fingerprint analysis

Qualitative analysis of the volatile components in DBJ and XBJ chrysanthemum was performed using the built-in NIST and IMS databases of the software. A comprehensive analysis yielded a total of 125 signal peaks, of which 66 compounds were tentatively identified, encompassing aldehydes, alcohols, esters, ketones, furans, and related categories. Detailed information is presented in Table 2, where numbers in the “Name” column indicate unidentified compounds.

**Table 2:** Qualitative results of GC-IMS.

No.	Compound	CAS	Formula	MW	Retention index (RI)	Retention time (Rt [s])	Drift time (Dt [a.u.])
1	Acetone	67 - 64 - 1	C3H6O	58.1	515.6	120.948	1.12101
2	2-Butanone	78 - 93 - 3	C4H8O	72.1	598.1	148.018	1.25034
3	1,8 Cineole - M	470 - 82 - 6	C10H18O	154.3	1038.7	667.872	1.29008
4	1,8 Cineole - D	470 - 82 - 6	C10H18O	154.3	1037.5	665.741	1.73049
5	1-Hexanol - M	111 - 27 - 3	C6H14O	102.2	872.5	367.624	1.32505
6	1-Hexanol - D	111 - 27 - 3	C6H14O	102.2	870.5	364.909	1.63659
7	(R)-Alpha-pinene - M	7,785 - 70 - 8	C10H16	136.2	936.6	469.876	1.21581
8	(R)-Alpha-pinene - D	7,785 - 70 - 8	C10H16	136.2	935.1	467.161	1.29066
9	(R)-Alpha-pinene - T	7,785 - 70 - 8	C10H16	136.2	934.2	465.352	1.67503
10	Beta-Pinene - M	127 - 91 - 3	C10H16	136.2	978.3	553.126	1.22593
11	Beta-Pinene-D	127 - 91 - 3	C10H16	136.2	977.9	552.221	1.29066
12	Beta-Pinene - T	127 - 91 - 3	C10H16	136.2	975.8	547.696	1.64266
13	Beta-Pinene - P	127 - 91 - 3	C10H16	136.2	975.4	546.791	2.19089
14	3-Methyl-2-butenal - M	107 - 86 - 8	C5H8O	84.1	781.9	264.255	1.09
15	3-Methyl-2-butenal - D	107 - 86 - 8	C5H8O	84.1	782.5	264.807	1.35951
16	(Z)-2-Pentenol	1,576 - 95 - 0	C5H10O	86.1	774.2	256.525	0.94754
17	Hexanal - M	66 - 25 - 1	C6H12O	100.2	796.9	279.162	1.2574
18	Hexanal - D	66 - 25 - 1	C6H12O	100.2	794.7	276.954	1.56134
19	Alpha-Phellandrene - M	99 - 83 - 2	C10H16	136.2	1008.1	613.375	1.21991
20	Alpha-Phellandrene - D	99 - 83 - 2	C10H16	136.2	1007.6	612.657	1.68542
21	d-Camphor - M	464 - 49 - 3	C10H16O	152.2	1125.8	850.866	1.33927
22	d-Camphor - D	464 - 49 - 3	C10H16O	152.2	1125.2	849.514	1.85261
23	Acetic acid ethyl ester	C141786	C4H8O2	88.1	628.6	159.489	1.33618
24	n-Octanal - M	124 - 13 - 0	C8H16O	128.2	1012.6	621.182	1.40443
25	n-Octanal - D	124 - 13 - 0	C8H16O	128.2	1012.6	621.182	1.82199
26	1-Penten-3 - one	1,629 - 58 - 9	C5H8O	84.1	690.4	185.983	1.32891
27	2-Methylpropanal	78 - 84 - 2	C4H8O	72.1	574.9	139.817	1.29759
28	2-Methyl-2-propenal	78 - 85 - 3	C4H6O	70.1	586	143.689	1.2304
29	Methyl acetate	79 - 20 - 9	C3H6O2	74.1	551.6	132.073	1.19717
30	2-Methyl-1-propanol	78 - 83 - 1	C4H10O	74.1	637.1	162.854	1.36911
31	1-Penten-3-ol	616 - 25 - 1	C5H10O	86.1	695.3	189.499	0.93935
32	Diethyl sulfide	352 - 93 - 2	C4H10S	90.2	713.1	202.916	1.04629
33	Acetoin - M	513 - 86 - 0	C4H8O2	88.1	718	206.786	1.06886
34	Acetoin - D	513 - 86 - 0	C4H8O2	88.1	718.7	207.302	1.3318
35	1-Pentanol-M	71 - 41 - 0	C5H12O	88.1	768.1	250.614	1.25311
36	1-Pentanol-D	71 - 41 - 0	C5H12O	88.1	768.1	250.614	1.51341
37	1-Pentanol-T	71 - 41 - 0	C5H12O	88.1	768.1	250.614	1.81149
38	Methyl 3-methylbutanoate - M	556 - 24 - 1	C6H12O2	116.2	777.9	260.162	1.19853
39	Methyl 3-methylbutanoate - D	556 - 24 - 1	C6H12O2	116.2	778.2	260.491	1.5316
40	4-Methyl-2-pentanol - M	108 - 11 - 2	C6H14O	102.2	759.1	242.054	1.2671
41	4-Methyl-2-pentanol - D	108 - 11 - 2	C6H14O	102.2	759.8	242.712	1.55399
42	(E)-2-Pentenal - M	1,576 - 87 - 0	C5H8O	84.1	758	241.066	1.10616
43	(E)-2-Pentenal - D	1,576 - 87 - 0	C5H8O	84.1	757.6	240.737	1.36367
44	2-Methylbutan-1-ol	137 - 32 - 6	C5H12O	88.1	743	227.567	1.47422
45	3-Methylbutan-1-ol	123 - 51 - 3	C5H12O	88.1	740.7	225.592	1.50081
46	Pentanal	110 - 62 - 3	C5H10O	86.1	707.5	198.595	1.42804
47	3-Methyl butanal	590 - 86 - 3	C5H10O	86.1	665.2	174.427	1.41378
48	2-Methyl butanal	96 - 17 - 3	C5H10O	86.1	671.5	177.12	1.38108
49	Furfural - M	98 - 01 - 1	C5H4O2	96.1	836.4	322.272	1.0852
50	Furfural - D	98 - 01 - 1	C5H4O2	96.1	836.4	322.272	1.33266
51	3-Methylbutyl acetate - M	123 - 92 - 2	C7H14O2	130.2	881.4	379.662	1.29838
52	3-Methylbutyl acetate - D	123 - 92 - 2	C7H14O2	130.2	881.8	380.149	1.74113
53	Heptanal - M	111 - 71 - 7	C7H14O	114.2	904.7	414.68	1.32968
54	Heptanal - D	111 - 71 - 7	C7H14O	114.2	903.5	412.734	1.6964
55	Propyl butanoate - M	105 - 66 - 8	C7H14O2	130.2	897.7	403.494	1.26558
56	Propyl butanoate - D	105 - 66 - 8	C7H14O2	130.2	898	403.98	1.68746
57	2-Heptanone - M	110 - 43 - 0	C7H14O	114.2	892.4	395.226	1.26409
58	2-Heptanone - D	110 - 43 - 0	C7H14O	114.2	891.8	394.253	1.63379
59	Propylsulfide	111 - 47 - 7	C6H14S	118.2	889	390.362	1.16421
60	(Z)-4-heptenal	6,728 - 31 - 0	C7H12O	112.2	901.1	408.844	1.15079
61	3-(Methylthio)propanal	3,268 - 49 - 3	C4H8OS	104.2	908.9	421.489	1.09415
62	Gamma - butyrolactone	96 - 48 - 0	C4H6O2	86.1	920.1	440.387	1.08462
63	Benzaldehyde - M	100 - 52 - 7	C7H6O	106.1	963.4	521.732	1.15292
64	Benzaldehyde - D	100 - 52 - 7	C7H6O	106.1	964.1	523.304	1.46859
65	(E)-2-Heptenal - M	18829 - 55 - 5	C7H12O	112.2	960.1	515.052	1.25814
66	(E)-2-Heptenal - D	18829 - 55 - 5	C7H12O	112.2	960.3	515.444	1.67349
67	Isobutyl isobutyrate - M	97 - 85 - 8	C8H16O2	144.2	917.3	435.672	1.31352
68	Isobutyl isobutyrate - D	97 - 85 - 8	C8H16O2	144.2	917.3	435.672	1.79902
69	2-Hexenal - M	505 - 57 - 7	C6H10O	98.1	854.7	344.502	1.17692
70	2-Hexenal - D	505 - 57 - 7	C6H10O	98.1	855.3	345.288	1.52027
71	3-Methylbutanoic acid - M	503 - 74 - 2	C5H10O2	102.1	841.9	328.784	1.22122
72	3-Methylbutanoic acid - D	503 - 74 - 2	C5H10O2	102.1	842.7	329.74	1.48922
73	2-Methylbutanoic acid - M	116 - 53 - 0	C5H10O2	102.1	849.7	338.327	1.20382
74	2-Methylbutanoic acid - D	116 - 53 - 0	C5H10O2	102.1	850.5	339.337	1.47229
75	Methyl pentanoate - M	624 - 24 - 8	C6H12O2	116.2	824.3	308.491	1.21771
76	Methyl pentanoate - D	624 - 24 - 8	C6H12O2	116.2	823.9	308.028	1.5612
77	Allyl isothiocyanate	57 - 06 - 7	C4H5NS	99.2	885.2	384.998	1.08935
78	6-Methyl-5-hepten-2-one	110 - 93 - 0	C8H14O	126.2	992.8	585.505	1.17928
79	Alpha-Terpinene - M	99 - 86 - 5	C10H16	136.2	1024.2	641.6	1.21664
80	Alpha-Terpinene - D	99 - 86 - 5	C10H16	136.2	1025.9	644.649	1.71866
81	Beta-mircene - D	123 - 35 - 3	C10H16	136.2	996.3	593.431	1.728
82	Beta-mircene - T	123 - 35 - 3	C10H16	136.2	996.9	594.651	2.17631
83	(R/S)-Linalool - M	78 - 70 - 6	C10H18O	154.3	1,097	785.513	1.2213
84	(R/S)-Linalool - D	78 - 70 - 6	C10H18O	154.3	1096.8	785.042	1.70019
85	Alpha-Terpinolene - M	586 - 62 - 9	C10H16	136.2	1085.6	761.002	1.21969
86	Alpha-Terpinolene - D	586 - 62 - 9	C10H16	136.2	1086.7	763.359	1.3116
87	Gamma-Terpinene - M	99 - 85 - 4	C10H16	136.2	1063.3	715.28	1.21646
88	Gamma-Terpinene - D	99 - 85 - 4	C10H16	136.2	1063.3	715.28	1.70825
89	(Z)-sabinene hydrate - M	546 - 79 - 2	C10H18O	154.3	1,072	732.721	1.21969
90	(Z)-Sabinene hydrate - D	546 - 79 - 2	C10H18O	154.3	1071.1	730.835	1.72276
91	(E,E)-2,4-octadienal - M	30361 - 28 - 5	C8H12O	124.2	1113.9	823.158	1.24439
92	(E,E)-2,4-octadienal - D	30361 - 28 - 5	C8H12O	124.2	1114.6	824.82	1.78106
93	Isopentyl 2-methyl butanoate-M	27625 - 35 - 0	C10H20O2	172.3	1104.5	802.113	1.41497
94	Isopentyl 2-methyl butanoate - D	27625 - 35 - 0	C10H20O2	172.3	1,104	801.005	2.00147
95	Alpha-Terpineol - M	98 - 55 - 5	C10H18O	154.3	1172.4	968.76	1.21789
96	Alpha-Terpineol - D	98 - 55 - 5	C10H18O	154.3	1173.8	972.382	1.29584
97	Alpha-Terpineol - T	98 - 55 - 5	C10H18O	154.3	1174.1	973.288	1.78251
98	Borneol - M	124 - 76 - 5	C10H18O	154.3	1154.5	921.671	1.21367
99	Borneol - D	124 - 76 - 5	C10H18O	154.3	1155.9	925.293	1.84782
100	Benzoic acid	65 - 85 - 0	C7H6O2	122.1	1201.2	1049.356	1.22421
101	1-Phenylethyl acetate	93 - 92 - 5	C10H12O2	164.2	1194.6	1030.339	1.05145
102	Bornyl acetate - M	76 - 49 - 3	C12H20O2	196.3	1262.5	1244.336	1.225
103	Bornyl acetate - D	76 - 49 - 3	C12H20O2	196.3	1,263	1,246	2.19005
104	Isopulegyl acetate	89 - 49 - 6	C12H20O2	196.3	1,290	1343.339	1.38085
105	Geraniol formate	105 - 86 - 2	C11H18O2	182.3	1300.3	1382.466	1.22424
106	Methyl 2-methoxybenzoate	606 - 45 - 1	C9H10O3	166.2	1,339	1539.185	1.2304
107	Longifolene	475 - 20 - 7	C15H24	204.4	1403.4	1841.33	1.45061
108	2-Butylfuran	4,466 - 24 - 4	C8H12O	124.2	899.7	406.649	1.178
109	1	-	-	-	771.5	253.906	1.41824
110	2	-	-	-	826.4	310.811	1.1311
111	3	-	-	-	815.5	298.75	1.1678
112	4	-	-	-	818.9	302.461	1.49368
113	5	-	-	-	896.4	401.494	1.11102
114	6	-	-	-	951	497.093	1.20963
115	7	-	-	-	947.5	490.386	1.69764
116	8	-	-	-	948.2	491.606	2.10393
117	9	-	-	-	970.3	536.116	1.32171
118	10	-	-	-	970.9	537.336	1.75602
119	11	-	-	-	1020.2	634.457	1.12573
120	12	-	-	-	1110.9	816.512	1.16006
121	13	-	-	-	1112.4	819.835	2.04555
122	14	-	-	-	1214.9	1090.106	1.21578
123	15	-	-	-	1215.8	1092.823	1.36747
124	16	-	-	-	1240.3	1169.797	1.21156
125	17	-	-	-	1242.2	1176.135	1.87942

The suffixes “Monomer,” “Dimer,” “Trimer,” and “Polymer” denote the monomer, dimer, trimer, and polymer of the same substance, respectively, while numerals indicate unidentified peaks.

To more accurately discern the variances in volatile components between DBJ and XBJ, a characteristic fingerprint analysis was conducted using the Gallery Plot module of the LAV software. As illustrated in Figure 8, the results of the study are evident. In the created fingerprint plot, each row signifies all signal peaks identified in a single sample, with each column denoting the signal peaks of the respective volatile component across diverse samples. This methodology provides straightforward and comprehensive access to the complete set of volatile components for each specimen, thereby enabling effective comparative analysis of volatile components across different specimens.

**Figure 8:**
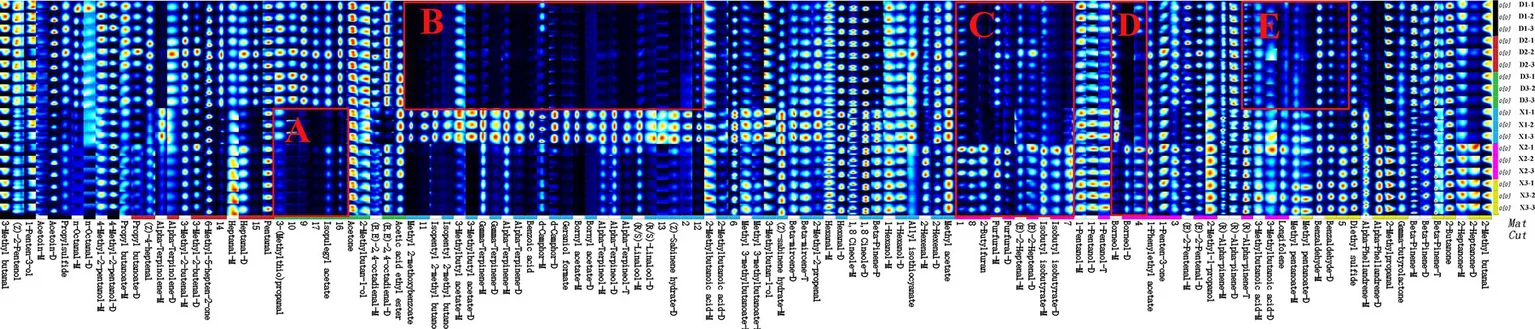
Characteristic fingerprint of volatile components in samples.

By comparing the volatile components between DBJ and XBJ, it was observed that the peak intensities (relative contents) of certain volatile organic compounds differed significantly. The relative content can be distinguished by the warmth of the spot color, with the sequence: dark blue < sky blue < yellow < orange < red. Figure 8 clearly reveals differences in volatile components among the sample groups. As shown in Region A, the contents of 3-(methylthio) propanal, isopulegyl acetate, Compound 9, Compound 10, Compound 16, and Compound 17 in DBJ were higher than those in XBJ. From Region B, it can be observed that the contents of alpha-terpinene - M, alpha-terpinene - D, gamma-terpinene - M, gamma-terpinene - D, d-camphor - M, d-camphor - D, alpha-terpineol - M, alpha-terpineol - D, alpha-terpineol - T, (R/S)-linalool - M, (R/S)-linalool - D, and (Z)-sabinene hydrate - D in XBJ chrysanthemum were higher than those in DBJ, whereas the contents of 3-methylbutyl acetate - M and Compound 12 were lower. Regions C and E indicate that the volatile compounds in these two regions were generally more abundant in XBJ than in DBJ, including isobutyl isobutyrate - M, isobutyl isobutyrate - D, (E)-2-heptenal - M, (E)-2-heptenal - D, furfural - M, furfural - D, 2 - butylfuran, methyl pentanoate - M, methyl pentanoate - D, longifolene, 3 - methylbutanoic acid - M, 3 - methylbutanoic acid - D, (R)-alpha-pinene - T, Compound 1, Compound 7, and Compound 8. Region D shows that the content of borneol - M in DBJ was higher than that in the XBJ, whereas the contents of borneol - D and Compound 4 were lower (19).

#### 3.3.3 GC-IMS combined with chemometric analysis

PCA is a common method for decreasing the dimension of data. It has the capacity to effectively reveal the intrinsic organization among samples while preserving the variance composition of the data to the best possible extent. OPLS-DA, a statistical methodology rooted in the tenets of partial least sums regression, finds common application in the reduction of data dimensionality and the identification of patterns. It efficiently handles multidimensional datasets and enables sample classification, discrimination, visualization, and future trend prediction.

Subsequently, the signal peak volumes extracted from the samples were incorporated into the SIMCA 14.1 software suite for PCA and OPLS-DA analysis. The outcomes of these analyses are presented in. As illustrated in Figure 9A (PCA score plot), the leading principal component manifested a contribution rate of 63.6%, while the secondary principal component manifested a contribution rate of 28.8%, thereby yielding a cumulative rate of 92.4%. This covered the majority of the data information from the samples, sufficiently reflecting the primary information in DBJ and XBJ samples. From Figure 9B (OPLS-DA score plot), it can be observed that DBJ and XBJ were well separated into two distinct groups, consistent with the PCA results. The dissimilarities between the DBJ and XBJ samples were pronounced, with an absence of regional overlap. Nevertheless, a certain degree of variability was identified within the XBJ sample group, which might be attributable to variables such as harvest time and processing techniques.

**Figure 9:**
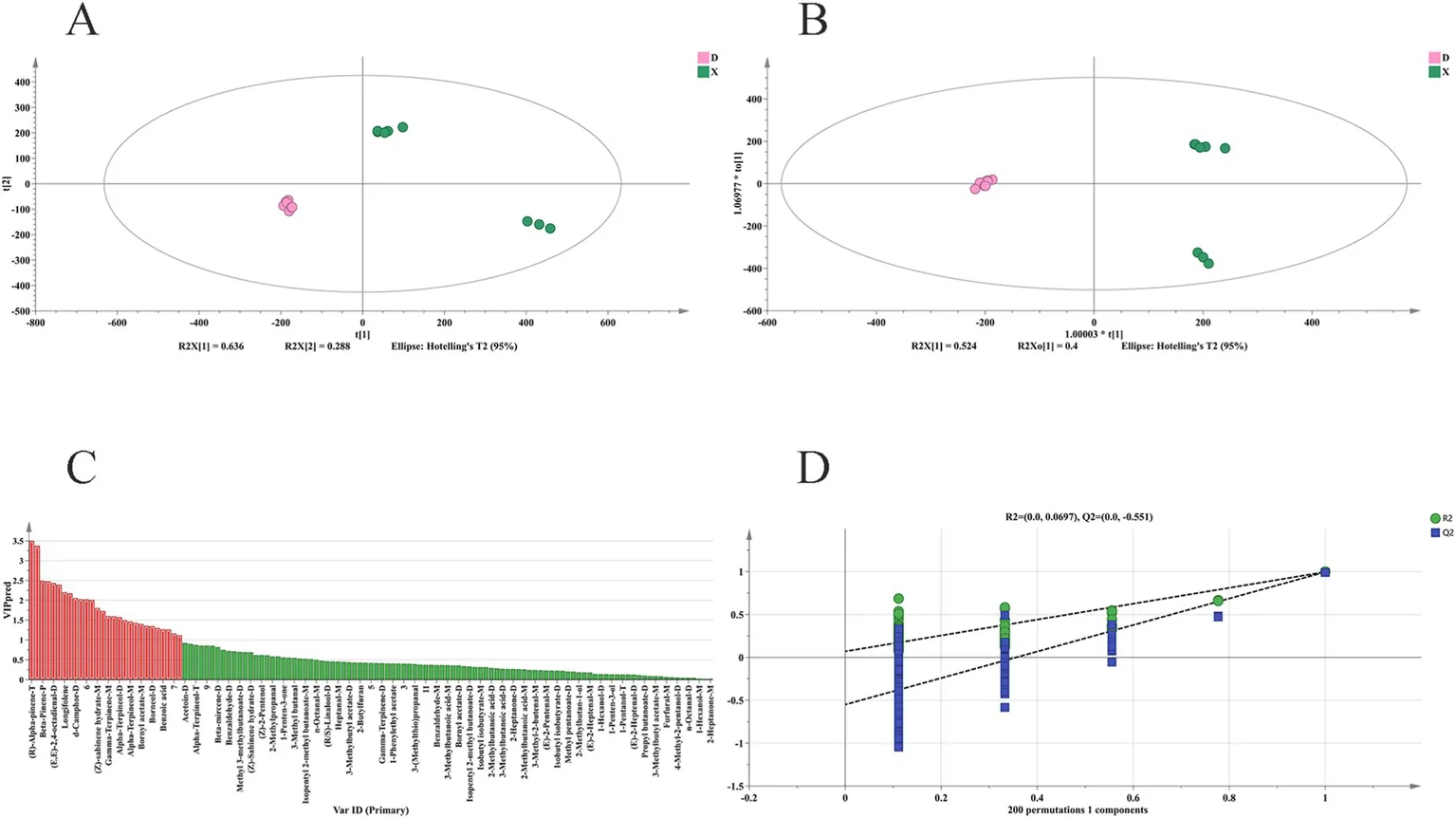
Chemometric analysis of GC-IMS measurements: **(A)** PCA Score plot. **(B)** OPLS-DA Score plot. **(C)** VIP Plot. **(D)** Permutation test plot with 200 permutations.

In the OPLS-DA model, the VIP value is frequently employed for the screening of key variables, as it quantifies the impact of each variable on the model’s classification. Higher VIP values are indicative of a more substantial contribution from that particular variable. Based on the criterion VIP > 1, Table 3 lists the volatile compounds with VIP > 1 in detail. As shown in Figure 9C and Table 3, 28 volatile compounds exhibited VIP > 1, namely (R)-Alpha-pinene - T, Compound 16, beta-pinene - P, alpha-phellandrene - D, (E,E)-2,4-octadienal - D, 1,8-cineole - D, longifolene, Compound 14, d-camphor - D, Compound 15, Compound 6, methyl 2-methoxybenzoate, (Z)-sabinene hydrate - M, (R/S)-linaloo - M, gamma-terpinene - M, 6-methyl-5-hepten-2-one, alpha-terpineol - D, alpha-terpinene - M, alpha-terpineol - M, Compound 17, bornyl acetate - M, alpha-phellandrene - M, borneol - D, beta-pinene - T, benzoic acid, isopulegyl acetate, Compound 7, and beta-pinene - M. This indicates that these 28 volatile components exert a significant influence on sample classification and represent the primary potential marker compounds responsible for the odor differences between DBJ and XBJ, as shown in Figure 10. A 200 - time permutation test was conducted on the OPLS-DA model. As demonstrated in Figure 9D, the intercept of the Q^2^ regression line with the vertical axis was less than zero, indicating no evidence of overfitting and validating the model. Consequently, these outcomes can be utilized to differentiate between the volatile components of DBJ and XBJ. In conclusion, based on the differences in volatile component contents determined by GC-IMS, commercially available DBJ and XBJ can be effectively distinguished (20).

**Table 3:** GC-IMS VIP scores (VIP > 1).

Var ID (Primary)	M3. VIPpred
(R)-Alpha-pinene - T	3.49745
16	3.37239
Beta-Pinene - P	2.48692
Alpha-Phellandrene - D	2.47627
(E,E)-2,4-octadienal - D	2.42856
1,8 Cineole - D	2.38623
Longifolene	2.19984
14	2.17007
d-Camphor - D	2.05218
15	2.02329
6	2.02146
Methyl 2-methoxybenzoate	2.00524
(Z)-sabinene hydrate - M	1.80307
(R/S)-Linalool - M	1.72702
Gamma-Terpinene - M	1.59664
6-Methyl-5-hepten-2-one	1.59198
Alpha-Terpineol - D	1.57303
Alpha-Terpinene - M	1.49252
Alpha-Terpineol - M	1.46346
17	1.42754
Bornyl acetate - M	1.39957
Alpha-Phellandrene - M	1.3533
Borneol - D	1.34498
Beta-Pinene - T	1.29585
Benzoic acid	1.26304
Isopulegyl acetate	1.26024
7	1.16141
Beta-Pinene - M	1.11519

**Figure 10:**
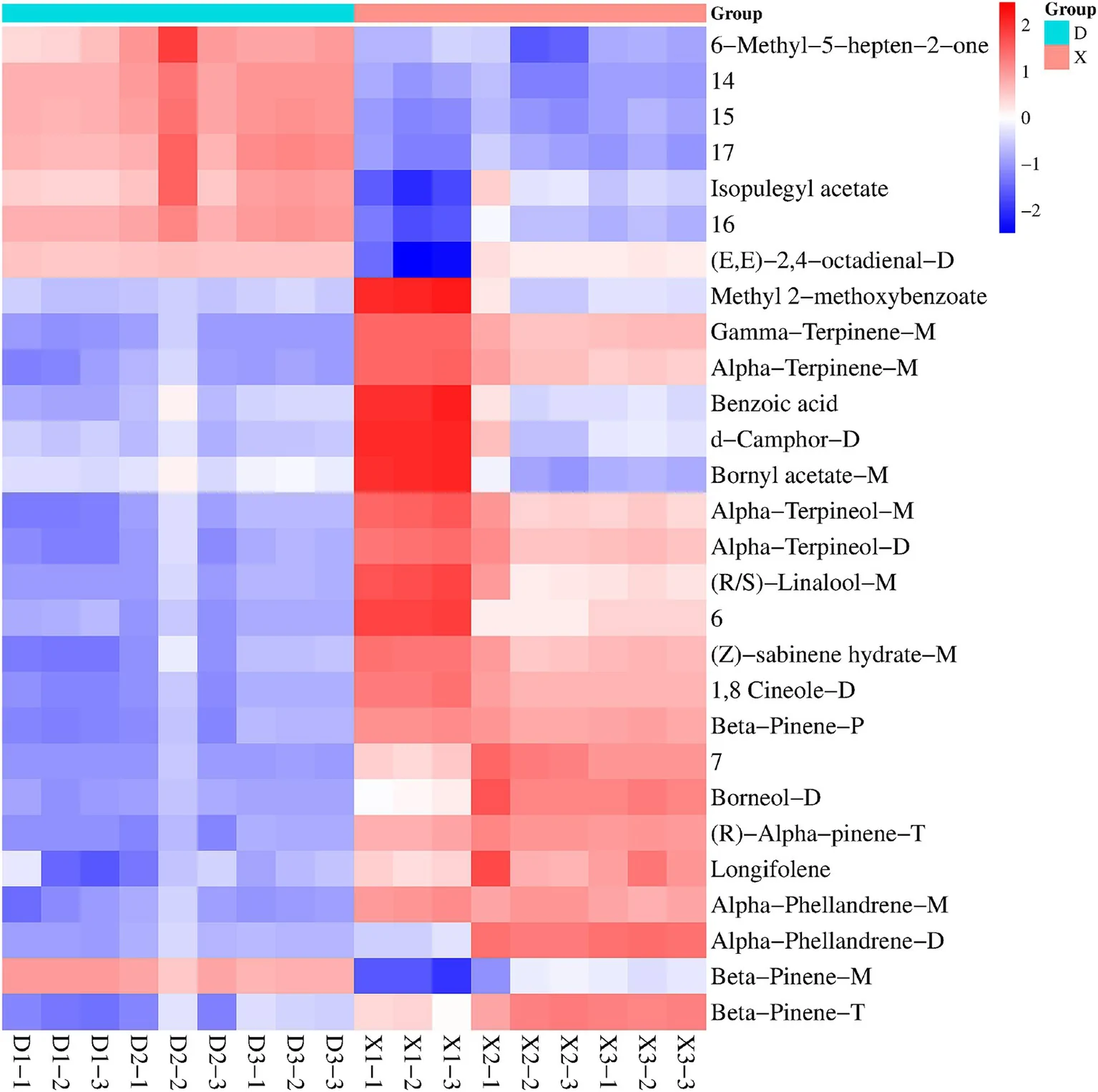
Heatmap of peak volumes of 28 volatile components (VIP > 1).

## 4 Discussion

The flavoromic divergence between DBJ and XBJ was mechanistically elucidated through the convergence of intelligent sensory perception and GC-IMS - derived volatile fingerprints (21). Principal component analysis of electronic nose data revealed distinct clustering of the two cultivars, with the W2W sensor (responsive to aromatic compounds and sulphur-containing volatiles) serving as the primary discriminator. This is consistent with previous findings that terpenoids constitute the dominant aroma backbone of chrysanthemum flower teas (22), implying that inter-cultivar variability in volatile organic compound stoichiometry drives sensor-specific responses. Such findings reinforce the discriminative validity of electronic nose profiling in authenticating chrysanthemum provenance and quality gradients (23).

GC-IMS facilitated a high-resolution visualization of volatile topographies, resolving 125 discrete signals corresponding to 66 annotated metabolites. The differential stoichiometry of monoterpenes, sesquiterpenes, and aldehydes—the phytochemical cornerstones of chrysanthemum’s medicinal bouquet—provided a molecular rationale for the observed E-nose signatures (24, 25). Notably, the spatiotemporal divergence in retention and drift kinetics substantiates that specific terpenoid biomarkers constitute the olfactory substratum detected by the sensors, aligning with the emergent utility of GC-IMS in architecting flavor fingerprints for food forensics (26). It should be noted that all volatile compounds in this study were tentatively identified based on library matching of retention indices and drift times against the built-in GC-IMS database (G. A. S., Germany), without confirmation by authentic standards.

Conversely, electronic tongue analysis revealed differential sensor response patterns between the two groups: DBJ elicited significantly stronger responses in the umami, bitter, and saltiness channels compared to XBJ, whereas XBJ exhibited comparatively attenuated response intensities across these taste-like sensors. These differential responses may hypothetically reflect variations in free amino acid pools, polyphenolic constituents, or inorganic cation profiles between the two cultivars (17, 27, 28); however, it should be noted that electronic tongue sensors provide cross-reactive, pattern-based outputs rather than compound-specific quantification. Therefore, these interpretations remain speculative and require validation through targeted chemical measurements (e.g., HPLC for free amino acids, ion chromatography for Na^+^/K^+^) or human sensory panels. Crucially, the multivariate triangulation of E-nose, E-tongue, and GC-IMS datasets via PCA and OPLS-DA engendered a robust discriminatory model that outperformed univariate analytical paradigms. This confirms that multi-modal data fusion serves as a powerful analytical heuristic for resolving morphologically cryptic ‘Boju’ cultivars. The observed differences between DBJ and XBJ should be interpreted as variations among commercial batches rather than strictly cultivar-intrinsic distinctions. Given that samples were procured from different vendors with incomplete documentation of primary processing methods, the flavor discrepancies likely arise from a combination of factors—including genetic background, vendor-specific drying techniques, potential sulfur fumigation, and pre-analytical storage conditions—rather than cultivar alone. This limitation should be considered when extrapolating the present findings to broader cultivar-level conclusions. Prospective investigations integrating untargeted metabolomics with hedonic sensory panels are warranted to bridge chemosensory phenotypes with consumer perception, thereby facilitating the ontogeny of flavor-centric quality markers for functional chrysanthemum nutraceuticals.

## 5 Conclusion

In summary, integrated electronic nose, electronic tongue, and GC-IMS analysis revealed distinct flavor profiles between commercial DBJ and XBJ batches. Chemometric analysis identified differential volatile patterns, with terpenoid-related compounds among the key discriminators contributing to inter-group aromatic variation. Both groups exhibited similar taste-like sensor response patterns across bitterness, umami, richness, and sweetness channels; however, DBJ elicited stronger saltiness-like and bitter-aftertaste sensor responses compared to XBJ, representing the primary axis of differentiation between the two commercial specifications.

GC-IMS analysis yielded a total of 125 signal peaks, of which 66 compounds were tentatively identified and classified predominantly as aldehydes, alcohols, esters, ketones, and furans; 17 peaks remained unannotated. Subsequent OPLS-DA modeling isolated 28 discriminative volatile markers (VIP > 1) contributing to the differentiation between the two commercial groups. Notably, six of these markers (Compounds 6, 7, 14, 15, 16, and 17) could not be assigned putative identities based on library matching alone.

However, these findings are limited to the six commercial batches examined under the present experimental conditions and should not be generalized beyond this scope. The observed differences may reflect a combination of cultivar., vendor-specific processing, and storage factors, rather than cultivar alone. Future investigations should prioritize structural elucidation of the aforementioned unannotated compounds using high-resolution techniques such as HR-MS or GC × GC–MS. Furthermore, larger, independently validated sample sets—coupled with targeted quantification of bioactive constituents, safety assessment, and human sensory validation—are required to confirm these preliminary findings and to establish more robust differentiation frameworks for commercial chrysanthemum products.

## Data Availability

The original contributions presented in the study are included in the article/supplementary material, further inquiries can be directed to the corresponding author.
